# Mapping the therapeutic versatility of WHO essential medicines: a systematic analysis of off-label indications

**DOI:** 10.1177/20420986251386215

**Published:** 2025-11-24

**Authors:** Ramya Kateel, Shreya Hegde, Sadhana Holla

**Affiliations:** Department of Pharmacology, Kasturba Medical College, Manipal, Manipal Academy of Higher Education, Manipal, Karnataka, India; Department of Pharmacology, Kasturba Medical College, Manipal, Manipal Academy of Higher Education, Manipal, Karnataka, India; Department of Pharmacology, Kasturba Medical College, Manipal, Manipal Academy of Higher Education, Madhav Nagar, Manipal, Karnataka 576104, India

**Keywords:** drug repurposing, drug safety, evidence-based medicine, off-label use, WHO essential medicine list

## Abstract

**Background:**

Off-label prescribing represents an important aspect of clinical practice globally. However, there are limited data on the extent, evidence base and guideline support of off-label indications among the World Health Organization’s essential medicines list (WHO EML), which serves as a cornerstone for healthcare systems, especially in low- and middle-income countries.

**Objectives:**

This study aimed to systematically analyse the prevalence, therapeutic distribution, quality of evidence and inclusion in clinical guidelines of off-label uses for drugs listed in the 2023 WHO EML.

**Methods:**

We conducted a descriptive analysis of all medications on the WHO EML using UpToDate^®^ Lexidrug™ database. For each medication, we extracted FDA-approved indications, identified off-label indications, the level of evidence supporting each off-label use (Level A, B, C, or G) and inclusion status in clinical practice guidelines. Off-label indications were categorized across 24 therapeutic systems, and system-to-system transitions from labelled to off-label uses were mapped.

**Design:**

Descriptive cross-sectional observational study using a structured database analysis.

**Results:**

Off-label prevalence among the WHO EML was 60.0%. The most frequent off-label use was observed in infectious diseases (30.5%) and oncology (25.2%). Most off-label uses were supported by Level C (32%) and Level B (30%) evidence, while only 11% were backed by Level A evidence. Notably, 64% of off-label uses were included in clinical guidelines, though many lacked associated evidence levels in Lexicomp. The majority of off-label uses remained within the same therapeutic system, with limited cross-system transitions.

**Conclusion:**

This first comprehensive analysis of off-label uses across the WHO EML demonstrates widespread repurposing of essential medicines with variable evidence quality.

## Plain language summary

A study to understand how WHO essential medicines are used beyond their approved purposes as off-labeled indications. (Medicines are labeled for specific approved conditions, but off-label use means doctors also prescribe them for other health problems based on experience or evidence.)

Medicines listed by the World Health Organization (WHO) as ‘Essential Medicines’ are meant to treat specific, approved health conditions. However, in real-world practice, doctors often use these medicines for other conditions too, which is called ‘off-label use’. In our study, we looked closely at how WHO Essential Medicines are used beyond their official purposes. We grouped the different off-label uses into 24 broad health areas, such as heart diseases, infections, or mental health. We also tracked how medicines originally approved for one system (like the lungs) are being used for problems in another system (like the brain). Our findings show that many essential medicines have a wide range of uses in patient care, which could help guide future updates to essential medicine lists and treatment guidelines.

## Introduction

According to the World Health Organization (WHO), ‘Essential medicines are those that satisfy the priority health care needs of a population’. They are meant to be consistently accessible within well-functioning health systems, in suitable dosage forms, with guaranteed quality, and at costs that are affordable for both individuals and healthcare systems. The WHO essential medicines list (WHO EML) serves as a critical framework guiding national formulary development and healthcare policy globally. This list is updated every 2 years by WHO. The current list is the 23rd list of essential medicines, released by WHO in 2023. This revision added several important medications, including new cancer treatments, innovative antibiotics to address antimicrobial resistance and medications for non-communicable diseases that are becoming increasingly prevalent worldwide. The list is divided into core and complementary medicines, with core medicines addressing primary health conditions and complementary medicines requiring specialized care or infrastructure.^1^

Off-label prescribing is a practice where a drug is prescribed for conditions beyond its approved uses. The term ‘off-label’ does not inherently suggest that the use is inappropriate, illegal, ineffective, or unsafe. Off-label use of medications represents an important aspect of clinical practice, providing treatment options for conditions with limited therapeutic alternatives, rare diseases, and populations often excluded from clinical trials.^2^ Off-label prescribing is a widespread practice globally, with significant variation across patient populations and drug classes. Studies report off-label prescription rates ranging from 11% to over 56%, influenced by factors such as patient age, drug type and regional prescribing practices.^3–8^ The prevalence is notably higher in fields such as oncology,^9,10^ paediatrics^11,12^ and cardiology^13^ where treatment options may be limited or evolving rapidly. In many cases, off-label use is driven by emerging clinical evidence, expert consensus or urgent patient needs that precede formal regulatory approval. While off-label prescribing can provide therapeutic flexibility and innovation, it also raises concerns regarding the strength of supporting evidence, patient safety and legal or ethical implications.

The WHO list of essential medicines is intended to be universally available and forms the backbone of public health systems. Off-label uses can fill gaps in treatment for conditions lacking approved therapies, particularly in low-resource settings. This will ensure maximizing the existing infrastructure and budgets. While off-label prescribing can offer valuable therapeutic options, it is often inadequately supported by data. Unsupervised off-label prescribing poses safety concerns. Hence, we planned to systematically analyse the prevalence, therapeutic distribution, evidence quality and guideline inclusion of off-label indications for WHO EML 2023. Understanding these patterns is essential for ensuring the safe, effective and rational use of essential medicines worldwide. Moreover, the findings can guide policymakers, healthcare providers and researchers in prioritizing areas where stronger evidence or clearer guidelines are needed to support patient care and global health goals. The WHO EML prioritizes cost-effective therapies, but studying off-label applications ensures these medicines can address broader health challenges without requiring costly new approvals.

## Methodology

### Data source and selection

We conducted a comprehensive analysis of off-label uses for all medications on the WHO EML 2023. UpToDate^®^ Lexidrug™ database^14^ served as our primary data source for identifying both labelled and off-label indications. We systematically searched each medicine included in the index of the WHO EML individually in the UpToDate^®^ Lexidrug™ database. Medications that lacked FDA approval were excluded from our analysis due to unavailability of standardized information in UpToDate^®^ Lexidrug™. Data were collected between January and March 2025. As the Lexidrug™ database primarily references FDA approvals, off-label designations were defined using FDA criteria.

In order to validate the reliability of UpToDate^®^ Lexidrug™ data for off-label indications, we randomly selected 10% of all EML-listed drugs with identified off-label indications for cross-checking. For these, we manually verified the presence or absence of the indication in the FDA-approved labelling via DailyMed.^15^ If the indication was not present in DailyMed, we classified it as off-label. Subsequently, we performed structured PubMed searches (using the drug name and indication) to identify supporting clinical studies or reviews, ensuring robust confirmation of off-label status and evidence.

Our validation analysis showed 100% concordance. All drugs in the validation sample (10% of the WHO EML) had off-label indications that were not listed in the FDA-approved labelling and were supported by relevant clinical evidence identified in PubMed. This yielded a 100% validation rate within the selected sample.

### Data extraction

For each included medication, we extracted the following data: All FDA-approved (labelled) indications, all identified off-label indications, level of evidence supporting each off-label use and inclusion status of off-label indications in clinical practice guidelines (level G). All these data were extracted for adult users.

The level of evidence supporting off-label uses was categorized based on UpToDate^®^ Lexidrug™ classifications: Level A indicates strong evidence from well-conducted trials or overwhelming historical data; Level B reflects moderate evidence from clinical trials with limitations or strong data from other designs; Level C includes observational studies or expert opinion with uncertain estimates; and Level G signifies off-label uses endorsed by at least one evidence-based or consensus clinical guideline. (Refer to Supplemental Table 1 for detailed definitions.)

### Therapeutic classification

Both labelled and off-label indications were systematically categorized according to the body system or therapeutic area they primarily affect. We employed a comprehensive classification system consisting of 24 categories (Supplemental Table 2).

For each medication, we documented the total number of off-label indications and categorized drugs based on the quantity of off-label uses (0, 1–10, 11–20, 21–30).

In addition to categorizing off-label uses, we documented therapeutic transitions, from their approved labelled uses to off-label applications in different physiological systems

### Statistical analysis

We performed descriptive statistical analysis using Jamovi 2.3.28 software (The Jamovi Project 2025). The results were presented using descriptive statistics, including frequencies, percentages and graphical representations to illustrate patterns in off-label use, evidence quality and guideline inclusion across therapeutic categories.

The reporting of this study conforms to the Strengthening the Reporting of Observational Studies in Epidemiology (STROBE) statement for observational research.^16^

## Results

We searched UpToDate^®^ Lexidrug™ for off-label indications of all medicines included in the WHO EML. Of the total medications listed, we were able to retrieve information for 486 drugs from UpToDate^®^ Lexidrug™. The remaining drugs (23) were excluded from our analysis as they lacked FDA approval, and consequently, information was not available in UpToDate^®^ Lexidrug™.

**Figure 1.**
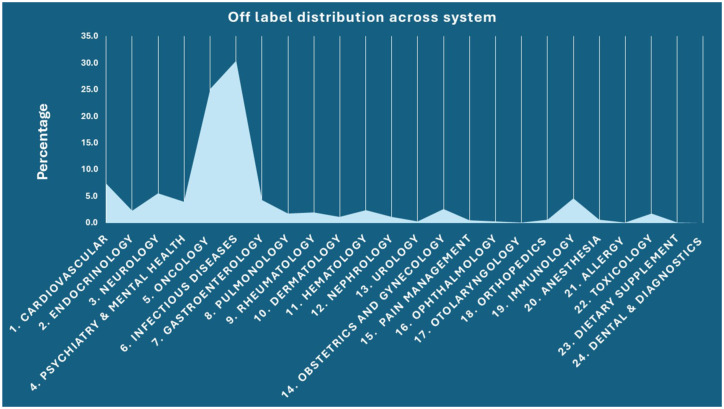
Percentage distribution of off-label use across different body system. This bar graph displays the percentage distribution of off-label indications among the WHO essential medicines across 24 therapeutic systems. The highest prevalence of off-label use was observed in infectious diseases (30.5%) and oncology (25.2%). Other systems such as urology, pain management, otolaryngology, ophthalmology, orthopaedics, anaesthesia, dietary supplements, dental and diagnostics contributed less than 1% each, highlighting the concentrated nature of off-label use within a few high-burden medical domains.

Among the 486 WHO essential medicines analysed, we identified 292 drugs (60.0%) with documented off-label uses. These off-label indications spanned across different therapeutic categories, as shown in Figure 1, with the most common being infectious disease (30.5%) and oncology (25.2%). Urology, pain management, otolaryngology, ophthalmology, orthopaedics, anaesthesia, dietary supplements, dental and diagnostics had less than 1% of off-label use. Table 1 shows top 10 WHO EML drugs with highest off-label uses. All the top 10 drugs were under oncology and infectious disease category. Rituximab (31) was the drug with the highest number of documented off-label use documented in UpToDate^®^ Lexidrug™ among the WHO EML.

**Table 1.** Top 10 WHO essential medicines with the most off-label uses.

Drug name	Number of off-label use	Therapeutic categories
Rituximab	31	Oncology
Cyclophosphamide	30	Oncology
Sulfamethoxazole + Trimethoprim	29	Infectious disease
Cisplatin	28	Oncology
Methotrexate	28	Oncology
Carboplatin	25	Oncology
Azithromycin	25	Infectious disease
Ceftriaxone	23	Infectious disease
Doxycycline	22	Infectious disease
Etoposide	22	Oncology

**Figure 2.**
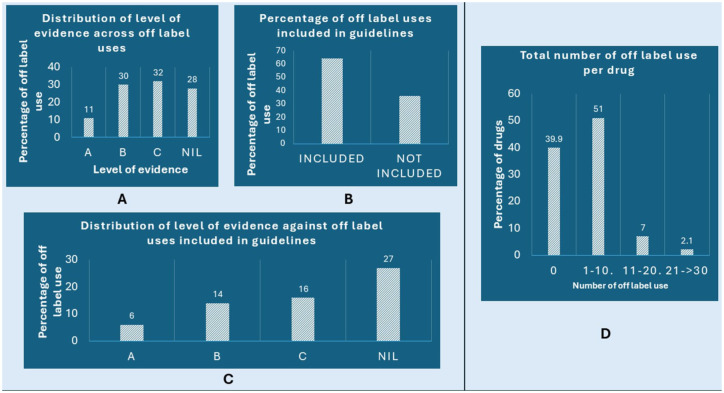
Comprehensive overview of the evidence supporting off-label use of the WHO essential medicines. (a) Illustrates the level of evidence supporting off-label indications across the analysed medications. (b) Quantifies the percentage of WHO essential medicines with off-label indications that have been formally incorporated into clinical practice guidelines. (c) Level of evidence for off-label uses that are also included in clinical guidelines. (d) Categorizes the WHO essential medicines based on the total number of identified off-label indications per drug.

As illustrated in Figure 2, the analysis of evidence and guideline support for off-label uses revealed several important trends. The majority of off-label indications (Figure 2(a)) were supported by Level C evidence (32%), indicating a heavy reliance on observational studies, case series or unsystematic clinical experience. This was followed by Level B (30%), with relatively few off-label uses backed by Level A (11%) evidence, which reflects well-conducted randomized controlled trials. Twenty-eight percent of the off-label uses did not have any specified level of evidence but were included in clinical guidelines, as per the UpToDate^®^ Lexidrug™ database.

In terms of clinical guideline inclusion (Figure 2(b)), 64% of off-label uses were found to be incorporated into established practice guidelines. Even among these, Figure 2(c) shows that most were supported by Level B (14%) or C (16%) evidence, and only 6% were supported by Level A evidence.

Figure 2(d) highlights the distribution of the number of off-label uses per drug. A significant number (51%) of WHO essential medicines had between 1 and 10 off-label indications, while fewer drugs had a high number of off-label uses (11–20 or 21–30).

**Figure 3.**
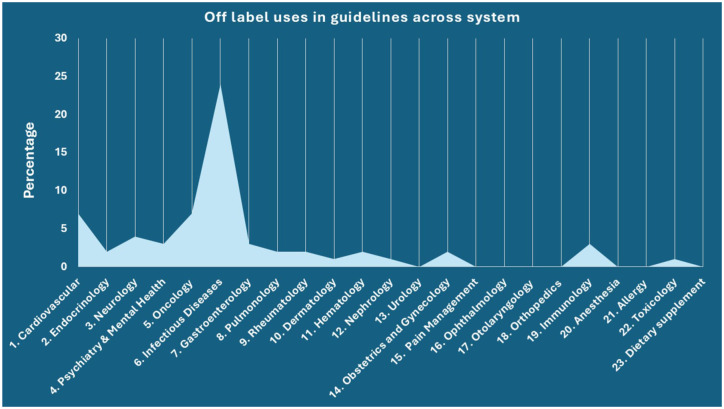
Distribution of off-label use included in guidelines in different system. This chart illustrates the proportion of off-label indications included in clinical guidelines, stratified by therapeutic system. Infectious diseases had the highest share of guideline-endorsed off-label uses (24%), followed by cardiovascular (7%) and oncology (7%) categories.

As shown in Figure 3, the distribution of off-label uses that are included in clinical guidelines varied notably across therapeutic systems. The highest proportion of guideline-supported off-label uses was observed in infectious diseases (24%), cardiovascular (7%) and oncology (7%). In contrast, all other therapeutic areas had either no off-label use included in guidelines or it varied between 1% and 4%.

**Figure 4.**
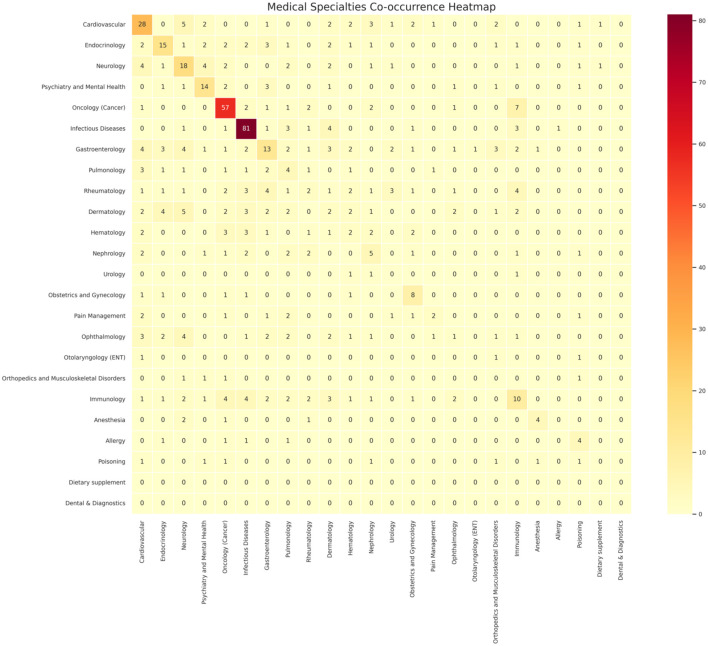
System-to-system transition of drug indications from labelled to off-label uses. This heat map visualizes the therapeutic shifts of WHO essential medicines from their approved (labelled) indications to off-label uses across different physiological systems. Each row represents the original therapeutic system of the drug’s labelled use, and each column represents the system in which the drug is used off-label. Darker shades indicate a higher number of transitions between the two systems. The majority of off-label uses remained within the same therapeutic system, particularly in infectious diseases, oncology, cardiovascular medicine, neurology and endocrinology. Cross-system transitions were relatively infrequent and primarily observed in a few immunological or complex multi-system drugs.

We also examined the therapeutic transition of drug indications, whether off-label uses remained within the same system as the labelled indication or crossed into a different therapeutic domain. As illustrated in the heat map in Figure 4, system movement is represented by colour intensity, with darker shades indicating a higher number of transitions between systems. The analysis revealed that most off-label uses occurred within the same therapeutic system as the original labelled indication, suggesting that drugs are frequently repurposed for conditions closely related to their approved use. This pattern was most prominent in infectious diseases, oncology, cardiovascular medicine, neurology and endocrinology, which showed the highest number of off-label uses confined within the same system. Conversely, cross-system transitions, where a drug is used off-label in a completely different therapeutic area, were relatively rare and seen in some of the immunology off-label uses.

## Discussion

Our comprehensive analysis of the WHO EML revealed several significant findings regarding off-label medication use. We found that 60% of the WHO essential medicines have at least one documented off-label use, demonstrating the widespread nature of this practice. Regarding evidence quality, only 11% of these off-label indications were supported by high-quality (Level A) evidence, while the majority relied on Level C (32%) or Level B (30%) evidence. Notably, 64% of off-label uses were incorporated into clinical practice guidelines; among them, only 6% were backed by Level A evidence. The therapeutic distribution analysis showed infectious diseases (30.5%) and oncology (25.2%) had the highest prevalence of off-label applications. Similarly, guideline-supported off-label uses were most common in infectious diseases (24%), followed by cardiovascular and oncology (7% each). Most drugs had between 1 and 10 off-label indications, with rituximab (31) having the highest number among all WHO essential medicines. Our system transition analysis revealed that most off-label applications occurred within the same therapeutic system as the drug’s approved indication, particularly in infectious diseases, oncology, cardiovascular medicine, neurology and endocrinology, with minimal cross-system transitions observed.

Our findings highlight that off-label use is a widespread phenomenon even among medications designated as globally essential, with 60% of the WHO essential medicines having at least one documented off-label indication. These results align with previous studies reporting variable but significant rates of off-label prescribing across different clinical settings and populations. The concentration of off-label uses in infectious diseases (30.5%) is similar to previous studies, ranging from 1% to 94%.^17–19^ In infectious disease, high prevalence may be driven by the need to respond to emerging pathogens or antimicrobial resistance patterns. The prevalence of oncology drugs’ off-label use in our study aligns with findings from previous research. A systematic review^9^ reported off-label use in oncology ranging from 18% to 41%, while our analysis found a prevalence of 25.2%. The increased off-label use is likely driven by the need to address rare malignancies or refractory disease states where approved treatment options are limited. Although the prevalence of oncology drugs with off-label indications was high in our study, only 7% of these off-label uses were included in clinical guidelines. This may be due to the low level of supporting evidence or the exclusion of certain indications from guidelines despite high-level evidence. As noted by Zarkavelis et al.,^20^ Mellor et al.^21^ even when strong evidence exists, many off-label uses remain unendorsed in clinical practice guidelines. Our analysis of therapeutic transitions revealed that most off-label uses occur within the same system as the drug’s approved indication. This pattern suggests that clinicians tend to repurpose medications for mechanistically related conditions, leveraging known pharmacological properties for similar pathophysiological processes. This approach is potentially safer than cross-system applications, as it builds upon established understanding of drug mechanisms and safety profiles within a specific therapeutic domain.

The evidence supporting off-label use was predominantly of lower quality (Level C (32%) and Level B (30%)), with only 11% of off-label uses supported by high-quality (Level A) evidence. Previous studies have also shown a similar trend of lower levels of evidence for off-label uses.^22,23^ This finding underscores a critical gap between clinical practice and the availability of robust, high-quality evidence. Despite these limitations, 64% of off-label uses were included in clinical guidelines. This suggests that expert consensus often supports these applications despite limitations in formal evidence. Interestingly, even within guideline-supported uses, Level A evidence was low (6%). The predominance of Level C evidence (32%) indicates that many off-label prescribing decisions are based on observational studies, case series rather than rigorous clinical trials. This situation creates potential patient safety concerns and underscores the need for more robust research to validate these uses.

Our findings carry substantial implications for patient safety, particularly in high-risk therapeutic areas such as oncology and infectious diseases. Real-world studies have consistently demonstrated a higher incidence of adverse drug events (ADEs) associated with off-label prescribing. For example, Eguale et al.^24^ reported that the incidence of ADEs was significantly higher for off-label prescriptions (19.7 per 10,000 person-months) compared to on-label use (12.5 per 10,000 person-months), with an adjusted hazard ratio of 1.44 (95% CI: 1.30–1.60), underscoring the elevated risk profile of such practices. In oncology, the off-label or off-guideline use of anticancer drugs often targets patients with advanced disease and poor performance status, as demonstrated in a large real-world analysis of 165,912 patients by Liu et al.^25^ Their study found that off-guideline prescribing was more common in patients with later-line therapies, poor ECOG performance scores and at academic centres, where experimental approaches are more frequently pursued. However, although this approach reflects a clinical need to explore alternative therapies, it also raises ethical and legal concerns, especially when off-label prescriptions are not accompanied by proper informed consent. Some of the studies^26,27^ also noted that off-label use of anticancer drugs often occurs without sufficient clinical trial evidence and may lead to heightened toxicity, increased patient costs and complex medico-legal implications. Case reports also illustrate the risks of off-label prescribing.^28,29^ For example, at least 31 cases of new-onset seizures have been associated with the off-label use of tiagabine in patients without epilepsy.^28^

In infectious diseases, off-label antimicrobial prescribing has critical public health consequences. Misuse and overuse are key contributors to antimicrobial resistance, a major global health threat. Over 23,000 deaths annually in the United States alone are linked to antimicrobial resistance.^15,30^ One of the predominant drivers is the indiscriminate off-label use of antibiotics, often without microbiological confirmation or guideline support. In such scenarios, off-label prescribing not only endangers individual patient safety but also accelerates the global spread of resistant organisms. Therefore, any off-label use, particularly in antimicrobials, must be subject to stringent clinical justification and integrated into antimicrobial stewardship programmes.^30,31^

Our findings underscore the need for stronger regulatory frameworks and global guidance on the off-label use of medicines. While off-label prescribing provides much-needed therapeutic flexibility, especially in low-resource settings, it must be balanced with the principles of evidence-based medicine and patient safety. While regulatory bodies require robust evidence before approving new indications, clinical needs often demand more rapid adoption of promising treatments. This discrepancy results in widespread off-label prescribing that, while often clinically necessary, may lack the safeguards of formal regulatory review. In the United States, the FDA permits off-label prescribing by licensed clinicians but restricts pharmaceutical companies from promoting such uses unless formally approved.^32^ The European Medicines Agency (EMA) takes a similar stance, while physicians are allowed to prescribe off-label based on clinical judgment, EMA does not regulate off-label use directly, and national authorities oversee safety monitoring.^33^ The WHO also recognizes off-label use as a widespread necessity, particularly in paediatrics and rare diseases, and encourages countries to strengthen pharmacovigilance systems to monitor outcomes.^34^ However, most low- and middle-income countries lack dedicated policies, databases or institutional mechanisms to track off-label drug safety, dosage accuracy or outcomes.^35^ This regulatory vacuum may increase the risk of inappropriate use, adverse events and inequitable access to care. Strengthening national policies, incorporating off-label use into pharmacovigilance reporting systems and providing clear legal and ethical guidance for clinicians are essential steps to balance therapeutic flexibility with patient safety. The integration of real-world evidence platforms and regional harmonization of off-label prescribing norms could also help reduce discrepancies across health systems and improve global drug safety governance. Expansion of provisional approval mechanisms or creation of special pathways for evaluating new indications of essential medicines could help bridge this gap while maintaining appropriate safety standards.

Most existing studies on off-label drug use in the literature have predominantly focused on the paediatric population,^36–38^ where off-label prescribing is common due to the limited number of clinical trials in children. However, our study specifically examined off-label use in adult populations, which also warrants greater attention and prioritization in research and policy discussions. Importantly, we analysed new therapeutic indications rather than different routes, dosage forms or age. Earlier research has concentrated on off-label use within a single therapeutic system, typically focusing on high-burden areas such as cardiovascular diseases,^39,40^ oncology,^9,41^ infectious diseases %^17,42,43^ or psychiatric disorders.^44^ In contrast, our study analysed off-label use across all therapeutic systems, using the WHO EML as a foundation. This allowed for a comprehensive evaluation of off-label patterns across the full spectrum of clinical practice and enabled a unique system-to-system transition analysis. We selected the WHO EML because it serves as the foundation for national formularies in many countries, making these drugs among the most commonly prescribed worldwide. To our knowledge, this is the first study to systematically analyse off-label use patterns across the entire WHO EML.

### Limitations

This study has fewer limitations that should be considered when interpreting its findings. First, our reliance on UpToDate^®^ Lexidrug™ as the sole data source may have missed off-label uses or the level of evidence not documented in this database. However, UpToDate^®^ Lexidrug™ is widely recognized as a comprehensive and reliable clinical drug information resource, regularly updated and utilized by healthcare professionals worldwide. It undergoes rigorous editorial review processes to ensure the accuracy and completeness of information. A 10% random validation of off-label classifications using DailyMed and PubMed demonstrated 100% concordance. While no discrepancies were identified in our subset, we acknowledge that extremely rare annotation errors or recent label changes could still exist outside the sampled group, and broader validation across multiple sources would further enhance generalizability. Second, UpToDate^®^ Lexidrug™ classifications are based primarily on FDA approvals, meaning that in some countries, what we classified as off-label may actually be approved indications by local regulatory authorities. The heterogeneity in global regulatory approval processes presents a fundamental challenge in characterizing off-label use on an international scale. This variation arises from differences in local disease burdens, healthcare infrastructure, regulatory priorities and the availability of region-specific clinical evidence.^45–47^ While our reliance on FDA approval status to define off-label use may not capture the full scope of global regulatory landscapes, this approach offers a standardized and transparent framework for systematic analysis. Importantly, many countries consider FDA approvals as a benchmark for national regulatory decisions, especially in low- and middle-income settings where capacity for independent review may be limited. Although our analysis may over- or under-estimate the true prevalence of off-label use in specific regions, it provides a robust and reproducible baseline that future studies can build upon by integrating regulatory data from additional agencies. For the off-label uses listed as included in clinical guidelines but without a specified level of evidence in the UpToDate^®^ Lexidrug™ database, we did not independently verify whether these guidelines to assess provided supporting evidence for their inclusion. Due to the high number of such off-label uses and the extensive time required, it was not feasible for us to review each guideline individually. As a result, we cannot be certain whether all such off-label recommendations are fully supported by robust evidence or merely listed as consensus, expert opinion or low-quality evidence. This may have led to overestimation or underestimation of the actual evidence base for some off-label uses. Future research should consider systematically sampling or comprehensively reviewing the primary clinical guidelines to better assess the quality of evidence underpinning these recommendations. Furthermore, clinicians should exercise caution when considering off-label prescribing based on guideline inclusion and are advised to consult the original guideline documents to understand whether the recommendation is supported by high-quality evidence or primarily based on expert consensus, before initiating treatment. From a methodological perspective, we employed descriptive statistics rather than inferential statistical analyses. This approach is appropriate given the exploratory nature of our study. Since we analysed the complete set of WHO essential medicines (rather than a sample), descriptive statistics provide a complete picture of the population of interest without requiring statistical inference. For healthcare practitioners and policymakers, knowing the prevalence and distribution of off-label uses may be more immediately useful than *p* values.

## Conclusion

This comprehensive analysis of off-label uses of the WHO essential medicines reveals widespread repurposing of these drugs across diverse therapeutic areas, with variable evidence quality supporting these applications. These findings can inform WHO, national regulatory bodies, and global health organizations in shaping provisional approval frameworks, identifying priority areas for evidence generation and updating clinical guidance on essential medicines. Our analysis reveals numerous gaps where rigorous clinical trials or systematic reviews could help elevate lower-tier evidence to higher standards, especially for off-label indications that are included in guidelines. Future work should focus on strengthening the evidence base for common off-label applications, developing clearer guidelines for appropriate off-label prescribing and exploring regulatory innovations that can bridge the gap between formal approval processes and evolving clinical practice.

## Supplemental Material

sj-docx-1-taw-10.1177_20420986251386215 – Supplemental material for Mapping the therapeutic versatility of WHO essential medicines: a systematic analysis of off-label indications Supplemental material, sj-docx-1-taw-10.1177_20420986251386215 for Mapping the therapeutic versatility of WHO essential medicines: a systematic analysis of off-label indications by Ramya Kateel, Shreya Hegde and Sadhana Holla in Therapeutic Advances in Drug Safety

## Declarations

**Ethics approval and consent to participate:** Not applicable, as this study involved publicly available data and did not include human participants or identifiable personal information.

**Consent for publication:** Not applicable.

**Availability of data and materials:** All data analysed in this study are available from the UpToDate^®^ Lexidrug™ database, which is a subscription-based resource. Derived summary data are available from the corresponding author upon reasonable request.
