# Supplementary material for: Mapping the therapeutic versatility of WHO essential medicines: a systematic analysis of off-label indications
Source: Ther Adv Drug Saf. 2025 Nov 24;16:20420986251386215. doi: 10.1177/20420986251386215 (PMC12644437; doi:10.1177/20420986251386215)
Supplement: sj-docx-1-taw-10.1177_20420986251386215 – Supplemental material for Mapping the therapeutic versatility of WHO essential medicines: a systematic analysis of off-label indications [file sj-docx-1-taw-10.1177_20420986251386215.docx]

UpToDate® Lexidrug™ classifies the level of evidence into the following categories:[ 14]

| Level A: | Consistent evidence from well-performed randomized controlled trials or overwhelming evidence of another form (e.g., results of the introduction of penicillin treatment) supporting the off-label use. Further research is unlikely to change confidence in the benefit estimate. |
| --- | --- |
| Level B | Evidence from randomized controlled trials with important limitations (inconsistent results, methodological flaws, indirect or imprecise evidence), or very strong evidence from other research designs. Further research is likely to impact confidence in the benefit and risk estimate and may change the estimate. |
| Level C | Evidence from observational studies (e.g., retrospective case series/reports with significant impact on patient care), unsystematic clinical experience, or potentially flawed randomized controlled trials (e.g., when limited options exist for the condition). Any effect estimate is uncertain. |
| Level G | Use substantiated by inclusion in at least one evidence-based or consensus-based clinical practice guideline |

**Supplementary Table1: level of evidence classification by UpToDate® Lexidrug™**

| 1 | Cardiovascular | 13 | Urology |
| --- | --- | --- | --- |
| 2 | Endocrinology | 14 | Obstetrics and Gynaecology |
| 3 | Neurology | 15 | Pain Management |
| 4 | Psychiatry & Mental Health | 16 | Ophthalmology |
| 5 | Oncology | 17 | Otolaryngology |
| 6 | Infectious Diseases | 18 | Orthopaedics |
| 7 | Gastroenterology | 19 | Immunology |
| 8 | Pulmonology | 20 | Anaesthesia |
| 9 | Rheumatology | 21 | Allergy |
| 10 | Dermatology | 22 | Toxicology |
| 11 | Haematology | 23 | Dietary Supplement |
| 12 | Nephrology | 24 | Dental & Diagnostics |

**Supplementary Table 2: Classification of therapeutic system**
